# Prevalence and clinical features of bone morphogenetic protein receptor type 2 mutation in Korean idiopathic pulmonary arterial hypertension patients: The PILGRIM explorative cohort

**DOI:** 10.1371/journal.pone.0238698

**Published:** 2020-09-23

**Authors:** Albert Youngwoo Jang, Bo-Gyeong Kim, Sunkoo Kwon, Jiyoung Seo, Hyung Kwan Kim, Hyuk-Jae Chang, Sung-A Chang, Goo-Yeong Cho, Sang Jae Rhee, Hae Ok Jung, Kyung-Hee Kim, Hye Sun Seo, Kye Hun Kim, Jinho Shin, Jun Soo Lee, Minsu Kim, Young Jae Lee, Wook-Jin Chung

**Affiliations:** 1 Department of Cardiovascular Medicine, Gachon University Gil Medical Center, Incheon, Korea; 2 Gachon Cardiovascular Research Institute, Gachon University, Incheon, Korea; 3 Lee Gil Ya Cancer and Diabetes Institute, Gachon University, Incheon, Korea; 4 Division of Cardiology, Section of Cardiovascular Imaging, Department of Internal Medicine and Cardiovascular Center, Seoul National University Hospital, Seoul, Korea; 5 Division of Cardiology, Severance Cardiovascular Hospital, Yonsei University College of Medicine, Yonsei University Health System, Seoul, Korea; 6 Division of Cardiology, Department of Medicine, Heart Vascular Stroke Institute, Samsung Medical Center, Sungkyunkwan University School of Medicine, Seoul, Korea; 7 Cardiovascular Center, Seoul National University Bundang Hospital, Seongnam, Gyeonggi, Korea; 8 Department of Cardiovascular Medicine, Wonkwang University Hospital, Iksan, Korea; 9 Division of Cardiology, Department of Internal Medicine, Seoul St. Mary’s Hospital, The Catholic University of Korea, Seoul, Korea; 10 Division of Cardiology, Department of Internal Medicine, Sejong General Hospital, Bucheon, Korea; 11 Department of Cardiology, Soonchunhyang University Hospital, Bucheon, Korea; 12 The Heart Center of Chonnam National University Hospital, Gwangju, Korea; 13 Division of Cardiology, Department of Internal Medicine, Hanyang University Medical Center, Seoul, Korea; University of Alabama at Birmingham, UNITED STATES

## Abstract

**Background:**

Pulmonary arterial hypertension (PAH) is a progressive chronic disease with poor outcomes. One reason for poor prognosis is the lack of understanding regarding individual variability in response to treatment. Idiopathic PAH (IPAH) patients with bone morphogenetic protein receptor type 2 *(BMPR2*) mutations have distinct phenotypes that are crucial for individualized therapy but evidence regarding their prevalence and clinical features in the Korean population is lacking. Therefore, the present study aimed to screen Korean IPAH patients for *BMPR2* mutations and analyze their clinical phenotypes.

**Methods:**

We enrolled 73 unrelated IPAH patients for *BMPR2* mutation screening between March 2010 to November 2015 from 11 hospitals in Korea. Thirty-three lineal family members from 6 families of *BMPR2* mutation carriers were also screened.

**Results:**

Among 73 patients, 16 (22%) had *BMPR2* mutations. Mutation carriers were younger (27 vs. 47 years; p = 0.02) and had a higher mean pulmonary arterial pressure (mPAP) than non-carriers (64 vs. 51 mmHg; p<0.05). Of the 16 individuals with mutations, 5 deletion, 2 splice-site, 6 nonsense, and 3 missense mutations were found, among which, 9 were newly identified mutation types. Patients less than 30 years old had more *BMPR2* mutations (44 vs. 14%; p = 0.04) and a higher mPAP (64 vs. 50 mmHg; p = 0.04) compared with those equaled to or over 30 years old. There were no differences in hemodynamic profiles or the proportion of *BMPR2* mutation carriers between groups according to sex.

**Conclusion:**

The prevalence of *BMPR2* mutations in Korean IPAH patients was 22%. Mutation carriers were younger and had a poorer hemodynamic profile compared with the non-carriers.

**Clinical trial registration:**

Clinicaltrials.gov NCT01054105

## Introduction

Pulmonary arterial hypertension (PAH) is a rare and fatal disease characterized by pulmonary vascular cell proliferation and a sustained increase in mean pulmonary artery pressure (mPAP), leading to right heart failure and eventually death [1, 2]. Among the World Health Organization (WHO) types of pulmonary hypertension (PH), idiopathic PAH (IPAH) is in group 1 and includes sporadic and familial cases with or without known germline mutations [3, 4]. An altered transforming growth factor-beta (TGF-β) related receptor signaling via the bone morphogenetic protein receptor type 2 (BMPR2) is the most common germline mutation [3]. Bone morphogenetic proteins are part of a TGF-β superfamily cytokine group regulating growth and differentiation of bone and cartilage that affect various cell types [5]. BMPR2 is a type II receptor with a long carboxyl-terminal sequence following the intracellular kinase domain [6]. Mutations in the *BMPR2* gene result in loss of function and reduced downstream signaling [7]. Such mutations are prevalent in cases of IPAH, where 55% to 75% of individuals with familial history and up to 40% of individuals with idiopathic cases were found to be *BMPR2* mutation carriers [4, 8, 9].

Targeted therapy with the use of endothelin receptor antagonists, phosphodiesterase-5 inhibitors, and prostanoids have markedly improved the likelihood of survival [10]. Despite such advances, poor prognosis is a reality for most individuals, in part due to the lack of understandings regarding individual phenotypic variability [11, 12]. One of the most well-described and most prevalent examples of individual variability in IPAH is the *BMPR2* mutation [13–15]. Heterozygous *BMPR2* mutation carriers usually present 10 years earlier than non-carriers [14] and are associated with more severe hemodynamic deterioration at diagnosis [14–16], respond poorly to vasodilators [16, 17], and are at higher risk of lung transplantation, although evidence regarding survival is debatable [7, 18, 19]. Understanding the phenotypic variabilities of *BMPR2* mutations may be crucial for individualized treatment strategies to improve outcomes.

Although there have been several case reports regarding *BMPR2* mutations in Korean IPAH patients [20], there is no established nationwide Korean multi-center cohort to study *BMPR2* mutations to date. Therefore, this study aimed to screen Korean IPAH patients for *BMPR2* mutations and investigate the mutation prevalence, clinical characteristics, and hemodynamic profile of carriers.

## Materials and methods

### Subjects

The Effect of *BMPR2* Gene Mutations on Hemodynamic Response by Iloprost Inhalation in Pulmonary Arterial Hypertension (PILGRIM) cohort is a prospective, investigator-initiated, multi-institutional clinical trial (NCT01054105). The study protocol was reviewed and approved by the institutional review board of each participating center (GIRBA-2278; Gachon University Gil Medical Center, Seoul National University Hospital, Severance Cardiovascular Hospital at Yonsei University College of Medicine, Samsung Medical Center, Seoul National University Bundang Hospital, Wonkwang University Hospital, Seoul St. Mary’s Hospital of The Catholic University, Sejong General Hospital, Soonchunhyang University Hospital, Chonnam National University Hospital, and Hanyang University Medical Center).

The PILGRIM study was designed with two phases. The first phase was to evaluate the prevalence, hemodynamic features, and short-term outcomes of Korean individuals with IPAH. The second phase of the study will focus on the hemodynamic response to iloprost inhalation. Herein, we present the first phase of the PILGRIM study.

All patients gave informed consents for participation. Clinical data were acquired from electronic medical records. Telephone interviews were conducted for patients lost during follow-up. Consecutive patients diagnosed with IPAH at 11 participating hospitals were enrolled and followed between March 1, 2010, and November 30, 2015. The inclusion criteria were: (1) patients aged between 20 to 80 years; (2) those with WHO group I PH (mPAP >25 mmHg and pulmonary artery wedge pressure [PAWP] <15 mmHg) confirmed by right heart catheterization (RHC) or those who satisfied the echocardiographic criteria (peak pulmonary arterial pressure >40mmHg and mPAP >30mmHg); (3) previously diagnosed PAH patients refractory to conventional treatment excluding iloprost inhalation solution (Ventavis™), and; (4) able to undergo a low-intensity exercise test (bicycle or walking). Exclusion criteria were patients with PH belonging to WHO groups II-V and patients concurrently using other pulmonary artery vasodilators such as an inhaled nitric oxide (NO) or endothelin antagonists except for phosphodiesterase-5 inhibitors. Detailed inclusion and exclusion criteria are shown in S1 Table. Other test modalities such as pulmonary function testing, chest computed tomography, perfusion scan, human immunodeficiency virus blood tests, and connective tissue disease markers were used to rule out other specific causes among those without WHO group I PH.

### The definition of IPAH in the current study

**Fig 1:**
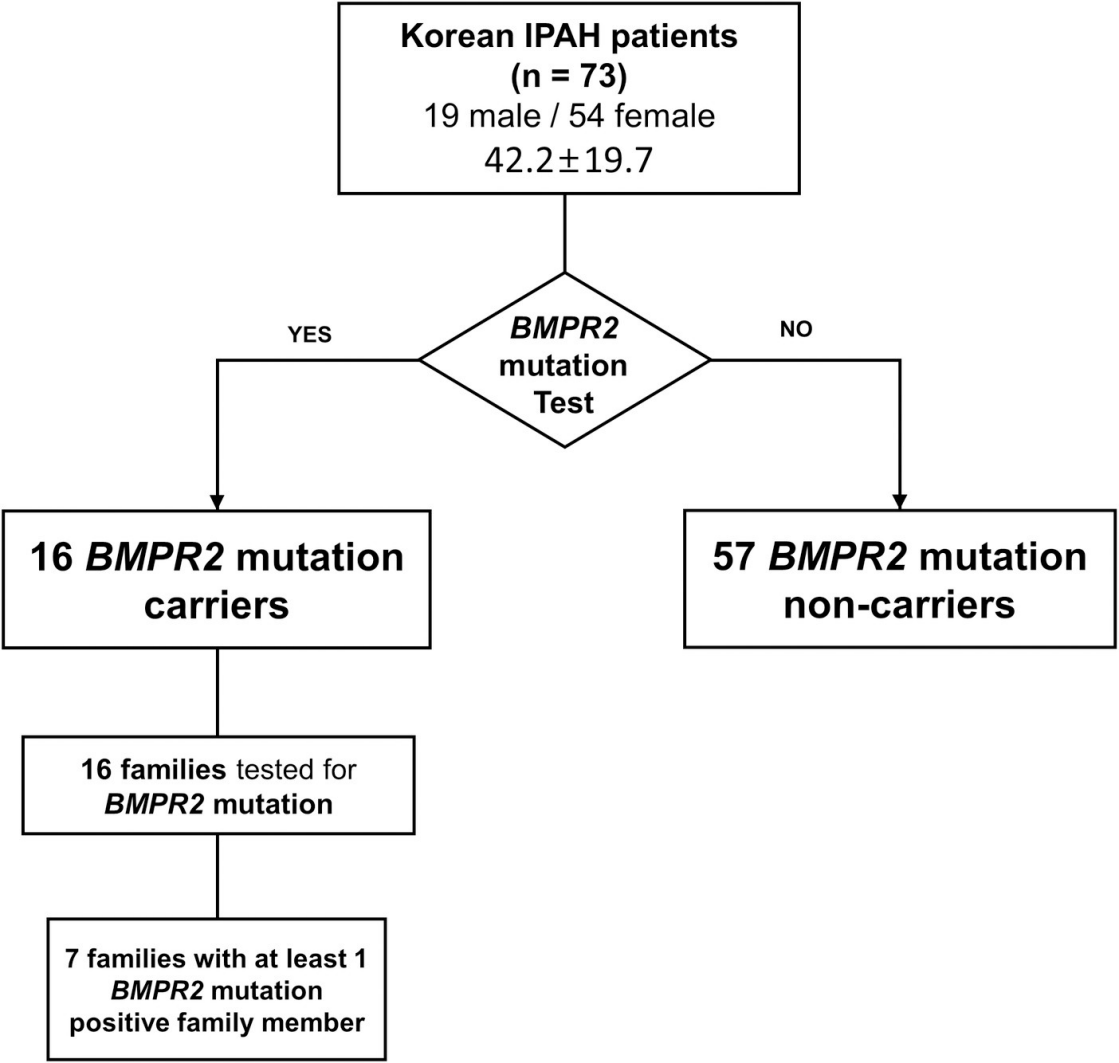
Diagram for detailed enrollment. The study population included 73 individuals with IPAH who were evaluated for *BMPR2* mutations. Sixteen individuals were positive for *BMPR2* mutation, among which 7 had at least one *BMPR2* mutation-positive family member. Fifty-seven individuals were *BMPR2* mutation carriers, whereas 17 were non-carriers. Abbreviations: PAH, pulmonary arterial hypertension; *BMPR2*, bone morphogenetic protein receptor type 2; IPAH, idiopathic pulmonary arterial hypertension.

In this study, IPAH was defined as individuals with WHO group I PH without apparent cause regardless of family history, which included all cases of sporadic or familial PAH. The sporadic and familial cases were not separately analyzed because they have indistinguishable histopathological and clinical phenotypes [21]. Additionally, *BMPR2*-associated PAH is an autosomal dominant disease with variable penetrance, indicating that all *BMPR2* mutation carriers have the heritable disease regardless of the clinical presentation of family members [13]. Thus, a total of 33 lineal family members of all *BMPR2* mutation-positive patients (33 lineal family members among 16 families of 16 mutation carriers) were examined. A diagram for enrollment is shown in Fig 1.

### Hemodynamic measurements

All diagnoses were confirmed by RHC. RHC was performed to measure hemodynamic values at initial diagnosis for all patients. The mPAP, PAWP, cardiac output, and pulmonary vascular resistance (PVR) were recorded.

### Molecular methods

Blood samples of patients were collected in vacuum blood collection tubes containing ethylenediaminetetraacetic acid. Genomic DNA was extracted from the blood or buffy coat samples using an Exgene Blood SV mini-kit (GeneAll, Korea) according to the manufacturer’s protocol. All exons and the flanking intronic sequences of the *BMPR2* gene were amplified by polymerase chain reaction using PrimeSTAR HS DNA Polymerase (TAKARA, Japan) and the appropriate primer sets. The primers used in this study are shown in S2 Table. Polymerase chain reaction products were purified using an Expin Combo GP kit (GeneAll, Korea). The purified samples were subsequently subjected to direct sequencing using a BigDye Terminator v3.1 cycle sequencing kit (Life Technologies, USA) and ABI 3730xl DNA Analyzer (Applied Biosystems, USA). The GenBank accession numbers for the reference sequences used in this study were NM_001204.6 for cDNA and NC_000002.12 for the genomic DNA of *BMPR2*.

### Literature search

We obtained cohort or meta-analysis data regarding individuals with IPAH with or without *BMPR2* mutations through a systematic search of the Cochrane Library, PubMed/MEDLINE, and Web of Science databases. The search terms were: 1) “BMPR2” OR “bone morphogenetic protein receptor type 2”; AND 2) “idiopathic PAH,” OR “heritable PAH” OR “sporadic PAH,” OR “familial PAH.” As a result, 11 studies contained relevant data with more than 50 patients enrolled.

### Statistical analysis

Data are presented as mean±standard deviation for normally distributed data, whereas continuous non-normally distributed data are given as a median and interquartile range (IQR). Discrete data are given as numbers and/or percentages. *P*-values less than 0.05 were considered statistically significant. The clinical and hemodynamic parameters between *BMPR2* mutation carriers and non-carriers were compared The Pearson χ^2^ test and Mann-Whitney U-test were used to compare normally and non-normally distributed data, respectively. The median follow-up was analyzed among those who did not die during follow-up. Mortality rates between groups were not analyzed due to a lack of statistical power. Statistical analyses were performed using SPSS v. 23.0 for Windows (IBM Corp., Armonk, NY, USA).

## Results

### Patient characteristics

We enrolled a total of 73 Korean patients diagnosed with IPAH from 11 health centers in Korea. Table 1 summarizes the demographic variables, functional/hemodynamic status, and drug regimen in *BMPR2* mutation carriers and non-carriers. The mean age at initial diagnosis regardless of etiology was 42.7±19.8 years and 27% were men. The mean age at diagnosis for carriers was lower than non-carriers by approximately 20 years (27.2±11.1 vs. 46.6±19.6 years; p = 0.022). The mutation carriers showed a higher mPAP than the non-carriers (63.7±17.3 vs. 50.5±15.2 mmHg; p < 0.05). Additionally, the PAWP in the carriers was significantly lower than in the non-carriers (6.8±2.2 vs. 10.8±3.5 mmHg; p = 0.020). Other hemodynamic profiles including PVR and cardiac output showed no significant differences between the two groups. The proportion of patients on PAH targeted therapy, including prostaglandin I_2_ (PGI_2_), phosphodiesterase-5 inhibitors, endothelin receptor antagonists, and tyrosine kinase inhibitors were similar between the two groups (Table 1). The median follow-up duration of the cohort was 1.5 years (IQR, 1.0 to 2.2) (Table 1). The median follow-up of the carrier and non-carrier groups was 1.0 and 1.3 years, respectively (p = 0.806). Zero carriers but 4 non-carriers died during follow-up (Table 1).

**Table 1:** Demographic and clinical characteristics of *BMPR2* carriers and non-carriers.

		*BMPR2* mutation		
	Total (n = 73)	Carrier (n = 16)	Non-carrier (n = 57)	*P-value*
***Demography***				
Men, n (%)	19 (26)	4 (25)	15 (26)	0.857
Age (y), mean±SD	42.2±19.7	27.2±11.1	46.6±19.6	0.022*
***Functional and hemodynamic status***				
NYHA functional class ≥II, n (%)	52 (71)	10 (63)	42 (74)	0.270
mPAP, mmHg	53.3±16.5	63.7±17.3	50.5±15.2	<0.001*
PAWP mmHg	15.8±12.3	6.8±2.2	10.8±3.5	0.020*
PVR, mmHg/L/min	10.2±8.9	11.5±9.6	9.9 ±8.8	0.722
Cardiac output, L/min	4.2±2.2	3.7±1.2	4.3±2.4	0.092
***PAH targeted therapy***				
Prostaglandin I_2_, n (%)	20 (27)	2 (13)	18 (32)	0.391
PDE5i, n (%)	14 (19)	3 (19)	11 (19)	0.854
ERA, n (%)	38 (52)	10 (63)	28 (49)	0.649
TKI, *n (%)*	*2 (3)*	0 (0)	2 (4)	0.587
***Follow-up data***				
Median follow-up years (IQR)^†^	1.5 (1.0–2.2)	1.0 (1.0–2.1)	1.3 (0.9–2.3)	N/A
Death, n (%)	4 (5)	0 (0)	4 (7)	N/A

*Abbreviations*: *BMPR2*, bone morphogenetic protein receptor type 2; SD, standard deviation; NYHA, New York Heart Association; mPAP, mean pulmonary arterial pressure; PAWP, pulmonary artery wedge pressure; PVR, pulmonary vascular resistance; PDE5i, phosphodiesterase-5 inhibitors; ERA, Endothelin receptor antagonists; TKI, tyrosine kinase inhibitors. IQR, interquartile range

*P < 0.05

^#^Mann-Whitney test

^†^ Median follow-up information was gathered among survivors only

A total of 33 lineal family members of the 16 IPAH *BMPR2* mutation-positive patients agreed to undergo screening examinations (Fig 1). One to five members in addition to the proband of each family were evaluated. Seven families had at least 1-member tested positive for the *BMPR2* mutation (S3 Table). Penetrance of the *BMPR2* mutation was not evaluated due to the lack of information regarding the clinical symptoms of each family member.

### *BMPR2* mutations

We found *BMPR2* mutations in 21.9% (16/73) of Korean individuals with IPAH (Table 2). Among the 16 mutations, 6 (37.5%) were nonsense, 5 (31.3%) were deletion, 2 (12.5%) were splice-site, and 3 (18.8%) were missense types. Six (37.5%) mutation types were previously reported [7, 22–24] and 2 nonsense mutations were identified in 2 different individuals. Of the 9 novel mutation types identified in the current study, 5 were deletion, 2 were splice site, one was nonsense, and one was missense (Table 2).

**Table 2:** Description of *BMPR2* mutation-positive genes.

Patient	Classification	Location	Mutation type	Nucleotide change	Protein change	Reference Number
1	idiopathic	exon 6	nonsense	c.631C>T	p.Arg211X	[10]
2	idiopathic	exon 8	nonsense	c.994C>T	p.Arg332X	[10]
3	idiopathic	exon 12	nonsense	c.2695C>T	p.Arg899X	[4]
4	idiopathic	5’UTR-exon1	deletion	c.1-1_8del	p.?	novel
5	idiopathic	exon 9	missense	c.1258T>C	p.Cys420Arg	[26]
6	heritable	exon 4	deletion	c.451del	p.Ile151fs	novel
7	idiopathic	exon 8	deletion	c.1042-1047del	p.Val348_Ile349del	novel
8	heritable	intron 5	splice site	c621+1G>T	p.?	novel
9	heritable	exon 8	deletion	c.1028del	p.Asn343fs	novel
10	heritable	intron6	splice site	c.853-2A>C	p.?	novel
11	heritable	exon12	nonsense	c.2695C>T	p.Arg899X	[4]
12	heritable	exon11	deletion	c.1448delGT	p.Cys483fs	novel
13	idiopathic	exon 6	nonsense	c.846T>G	p.Tyr282X	novel
14	idiopathic	exon 9	missense	c.1226T>C	p.Leu409Pro	novel
15	heritable	exon11	missense	c.1471C>T	p.Arg491Trp	[28]
16	idiopathic	exon 8	nonsense	c.994C>T	p.Arg332X	[10]

Abbreviation: UTR, untranslated region

### Subgroup analysis

Further stratified analysis was performed according to age and sex (Table 3). In patients under the age of 30, the proportion of *BMPR2* mutation carriers were significantly higher than the non-carriers (43.8% vs. 15.7%; p<0.001). The mean age difference between the younger and older groups was approximately 30 years. All deaths occurred in the older group (Table 3). There were no significant differences in age, hemodynamic profiles, or death between the sexes (Table 3).

**Table 3:** Demographic characteristics, *BMPR2* mutation status, hemodynamic profile, and clinical outcomes of subgroups.

**Age at diagnosis**			
	**< 30 (n = 16)**	**≥ 30 (n = 57)**	***P-value***
**Age (y), mean±SD**	20±7	50±17	<0.001
***BMPR2* mutation carriers, n (%)**	7 (43.8)	8 (14.0)	0.035
**mPAP, mmHg**	64±15	50±16	0.003
**PAWP mmHg**	20±12	15±13	0.314
**PVR, mmHg/L/min**	12±8	10±9	0.433
**Cardiac output, L/min**	3.9±1.1	4.3±2.4	0.652
**Sex**			
	**Male (n = 19)**	**Female (n = 54)**	***P-value***
**Age (y), mean±SD**	49±21	41±19	0.13
***BMPR2* mutation carriers, n (%)**	4 (21.0)	11 (20.0)	9.86
**mPAP, mmHg**	50±14	54±17	0.363
**PAWP mmHg**	15±10	16±13	0.882
**PVR, mmHg/L/min**	7.8±4.8	11.1±9.8	0.258
**Cardiac output, L/min**	5.1±2.4	3.9±2.1	0.093

Abbreviations: SD, standard deviation; *BMPR2*, bone morphogenetic protein receptor type 2; mPAP, mean pulmonary arterial pressure; PAWP, pulmonary artery wedge pressure; PVR, pulmonary vascular resistance

## Discussion

According to the present study, the prevalence of *BMPR2* mutations in Korean individuals with IPAH is 21.9%. In addition, Korean *BMPR2* mutation carriers are younger and have poorer hemodynamic profiles at diagnosis than non-carriers. Survival analysis was not performed in the current study due to the lack of statistical power.

The discovery of the association between PAH and *BMPR2* mutations has led to a better understanding of the pathobiology of PAH. *BMPR2* mutations increase the susceptibility to apoptosis in the endothelial cells and promote the proliferation of pulmonary arterial vascular smooth muscle cells [25]. These changes in the vasculature are thought to accelerate the development of PAH [26]. Previous studies report that *BMPR2* mutation carriers present at initial diagnosis at an earlier age and have more severe hemodynamic profile than non-carriers [14–16, 27]. The age of PAH diagnosis for the *BMPR2* gene mutation carriers has been reported to be 10 years earlier than in non-carriers, with the mean age ranging between 28 and 38.5 years [7, 13, 14, 16, 17, 19, 22–24, 28]. However, the present study data show that the *BMPR2* mutation carriers were diagnosed at the age of 27.9±11.4 years, which is approximately 10 years younger than the age of diagnosis in all international cohorts except for the Chinese [7, 13, 15–17, 19, 22, 24, 28, 29]. Thus, Korean *BMPR2* mutation carriers were 20 years younger than their non-carrier counterparts.

Despite the age gap between the two groups, *BMPR2* mutation carriers in the present study showed the highest gap of mPAP (13.2 mmHg) between carriers and non-carriers compared with other cohorts, including meta-analysis data, which only showed a 2 to 7 mmHg gap (Table 4) [7, 13, 15–17, 19, 22, 24, 28, 29]. Although the present study showed similar trends of higher mPAP at diagnosis in *BMPR2* mutation carriers compared to non-carriers, the uniquely high mPAP warrants further investigation. These results also indicate the importance of early screening for *BMPR2* for the initiation of aggressive treatment in patients to ensure better outcomes [10]. This has been previously suggested for the Japanese population which possesses similar genetic background [30].

**Table 4:** Comparison of clinical and hemodynamic features of *BMPR2* mutation carriers and non-carriers.

Country	PAH type	*BMPR2* mutation	Male/Female	Age, y	mPAP, mmHg	PVRI,	CI, L/min/m^2^	Familial cases, %	Reference
						mmHg/L/min/m^2^			
Korea	IPAH	Present (N = 16)	1/3.0	27.2±11.1	63.7±17.3	10.1±2.4	2.1±0.4	-	-
		Absent (N = 57)	1/2.8	46.6±19.6	50.5±15.2	8.3±1.1	2.3±0.4		
Japan	IPAH and HPAH	Present (N = 26)	1/4.8	35±13	57.7±14.5	19.1±7.3*	1.9±0.5	-	Isobe [28]
		Absent (N = 36)	1/1.8	34±11	55.7±15.1	16.9±9.0*	2.2±1.0		
Japan	IPAH and HPAH	Present (N = 18)	1/2.2	37.4±12.7	60.8±15.4	21.5±9.4	-	18	Kabata [19]
		Absent (N = 31)	1/1.7	25.9±11.3	58.8±12.0	18.6±8.6	-		
China	IPAH and HPAH	Present (N = 50)	1/1.3	28±2	67±6	17.1±2*	2.0±0.2	-	Liu [22]
		Absent (N = 255)	1/2.9	32±4	60±6	14.6±2*	2.3±0.2		
China	IPAH and HPAH	Present (N = 37)	1/2.2	27.2±9.9	60.2±15.3	17.3±8.0	2.6±0.9	6	Yang [24]
		Absent (N = 154)	1/1.7	31.6±10.5	54.9±14.7	13.0±5.2	3.0±0.9		
US	IPAH and HPAH	Present (N = 41)	1/2.2	36.1±1.4	58.6±1.7	18.1±2.0*	1.9±0.1	74	Austin [13]
		Absent (N = 106)	1/3.1	42±2.3	58.3±1.7	14.0±0.8*	1.8±0.2		
US	IPAH and HPAH	Present (N = 27)	1/1.0	37.1±12.0	60.7±10.5	14.6±5.1	2.1±0.6	18	Elliott [17]
		Absent (N = 40)	1/6.0	38.2±10.9	56.9±11.3	12.3±6.1	2.3±0.7		
US	IPAH and HPAH	Present (N = 23)	-	-	61±13	26±14	2.0±1.1	22	Rosenzweig [16]
		Absent (N = 124)	-	-	59±20	21±14	2.4±1.5		
France	IPAH and HPAH	Present (N = 68)	1/2.0	36.5±14.5	64±13	17.4±6.1	2.1±0.7	16	Sztrymf [15]
		Absent (N = 155)	1/2.6	46.0±16.1	56±13	12.7±6.6	2.5±0.7		
Germany	IPAH and HPAH	Present (N = 49)	1/1.9	38.5±11.8	62.6±9.9	28.8 ±9.6	1.7±0.3	10	Pfarr [14]
		Absent (N = 179)	1/3.0	45.8±11.3	53.4±12.2	18.8±8.4	2.1±0.5		
Meta-analysis	IPAH, HPAH, and anorexigen	Present (N = 448)	1/3.2	35±15	60.5±13.8	16.6±8.3*	2.1±0.7	15	Evans [7]
		Absent (N = 1102)	1/3.6	42±18	56.4±15.3	12.9±8.3*	2.5±0.9		

Abbreviations: *BMPR2*, bone morphogenetic protein receptor type 2; mPAP, mean pulmonary arterial pressure; PAWP, pulmonary artery wedge pressure; PVRI, pulmonary vascular resistance index; CI, confidence interval; IPAH, idiopathic pulmonary arterial hypertension; HPAH, heritable pulmonary arterial hypertension

*Pulmonary vascular resistance is presented due to the lack of data regarding pulmonary vascular resistance index (units: mmHg/L/min)

**Fig 2:**
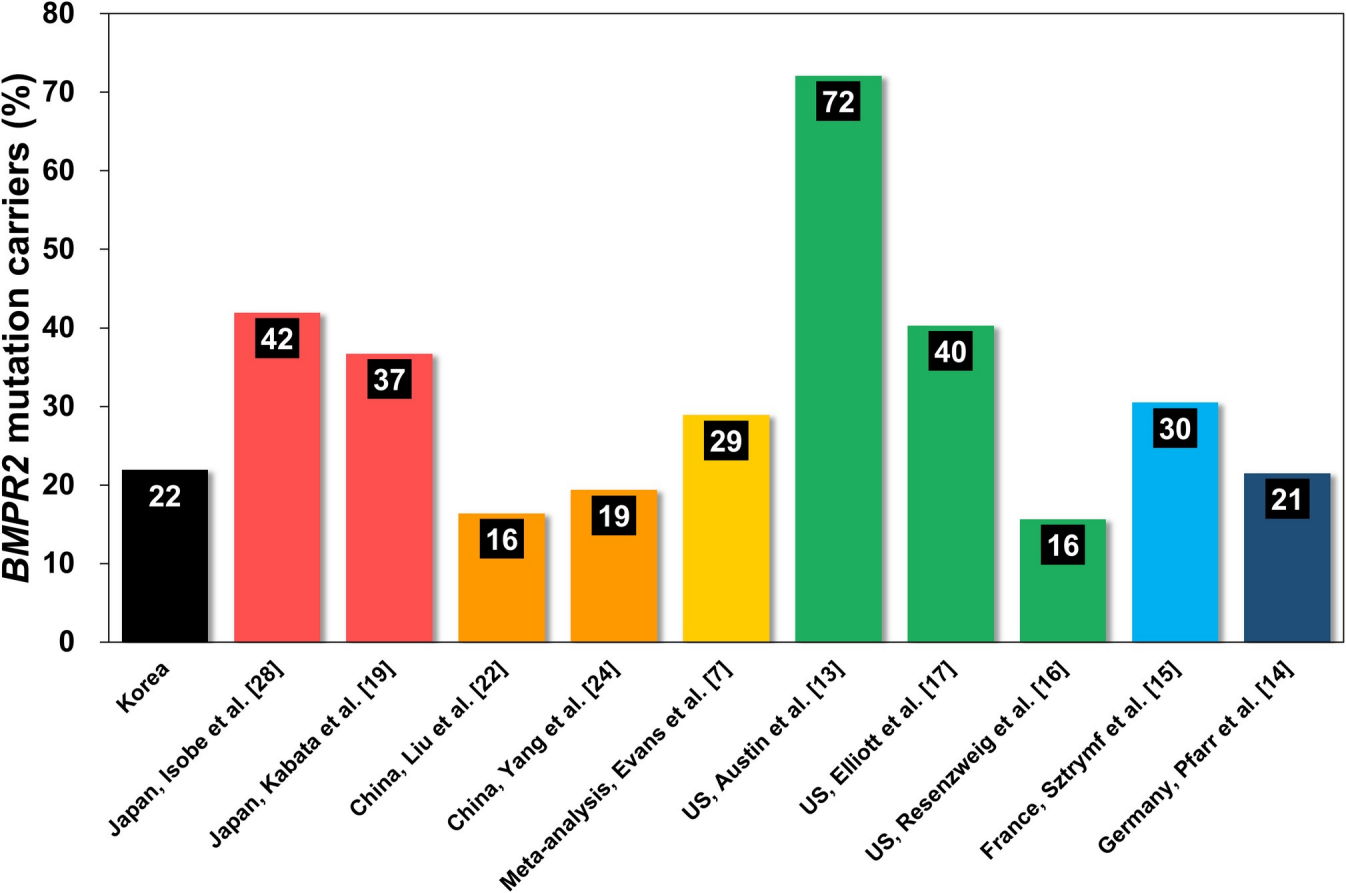
The worldwide prevalence between *BMPR2* mutation carriers and non-carriers in patients with idiopathic or heritable pulmonary arterial hypertension. The overall prevalence of *BMPR2* mutation carriers in IPAH patients in Korea was 22%, which is higher than in China and lower than in Japan. *BMPR2* = bone morphogenetic protein receptor type 2.

The prevalence of *BMPR2* mutations and the differences between characteristics of *BMPR2* carriers and non-carriers in the present study were similar to most cohorts in the literature, representing the United States (US), France, Germany, China, and Japan [7, 14–17, 19, 22, 24, 28], except for a single cohort from the US which mainly recruited familial cases (125 out of 169, 74%), as shown in Table 4 and Fig 2 [13]. All other cohorts enrolled familial cases to a similar degree (6 to 22%). The overall prevalence of *BMPR2* mutations in Korean IPAH patients was 22%, which is slightly higher than observed in Chinese and lower than in Japanese cohorts (Fig 2) [19, 22, 24, 28]. Data from Asian populations are limited, and the prevalence of *BMPR2* mutation differs between Japanese (36–41%) and Chinese (16–29%) studies (Fig 2) [19, 25, 27, 29]. This may be partly explained by the Chinese cohort having a relatively low percentage of familial cases (6%) compared with the Japanese (18%) [19, 24], considering that familial cases of PAH have a much higher prevalence of mutation carriers (70%) than sporadic cases (20%) [31]. It may also be suggested that the higher prevalence of *BMPR2* mutation carriers in Japanese cohorts may be due to the underestimation of familial cases because Asian patients are generally reluctant to share information about inherited diseases because they are considered a source of shame.

The percentage of patients carrying *BMPR2* mutations was significantly higher in patients younger than 30, compared with patients aged 30 or higher. This was consistent with the meta-analysis data, suggesting that *BMPR2* mutation may play a crucial role in the early development of PAH and severe hemodynamic symptoms [7]. Our study also shows that PAH patients were predominantly women (male:female, 1:2.8), although there was no difference in the proportion of *BMPR2* mutation carriers, hemodynamic profiles, or death according to sex [32].

The influence of *BMPR2* mutations on clinical outcomes has been a topic of controversy. Earlier studies reported that *BMPR2* mutation status does not affect overall survival or risk for lung transplantation [33]. A Japanese study found that the overall survival of *BMPR2* mutation carriers may be better than non-carriers due to ethnicity or the introduction of PGI_2_ infusion therapy [28]. On the other hand, a study with a Chinese cohort demonstrated that *BMPR2* mutation carriers had a significantly poorer survival, although the use of PGI_2_ or other medication was not specified [22]. According to a recent meta-analysis, mutation carriers had poorer outcomes especially in younger patients; however, information regarding comorbidities or PAH-targeted medication was not investigated [7]. Although we did not include a survival analysis in the present study due to the lack of statistical power, the *BMPR2* mutation carrier group had a higher percentage of deaths compared with the non-carrier group. It is most likely that this is by chance considering the small sample size and short follow-up. However, the trend is consistent with Japanese studies [10] that share a common east Asian heritage [34]. Also, the *BMPR2* mutation carriers in the current study were approximately 8 years younger than those in the meta-analysis [7].

The main limitation of this study was the small sample size and lack of information regarding family history. All 11 centers experienced difficulties enrolling patients, as the majority of the patients and family members were reluctant to share their genetic information and symptoms, which can be used against them in Korean society. In Asian societies, sharing information regarding heritable diseases is taboo because it is generally perceived as a shame. Therefore, we were not able to gather hemodynamic profiles or data on symptoms from all family members, hampering the effort to include individuals with heritable PAH. The Genetic Information Nondiscrimination Act (GINA) is a law that protects individuals from being discriminated against by insurance companies or employers after participating in research or genetic testing [35]. As Korean society has no protective measures against genetic discrimination, such as the GINA in the US, fear of being discriminated against dissuaded many patients from participating in this study. Legislative initiatives for genetic nondiscrimination are necessary for the continuation of genetic research in Korea. Another limitation of the study was that the phenotypic expression of each genetic mutation was not investigated. Future studies involving multi-omics data and deep phenotyping in Korean individuals with PAH are warranted [11].

## Conclusions

In the PILGRIM study, the *BMPR2* mutation carriers were 20 years younger and had higher mPAP than the non-carriers. Despite these discrepancies in baseline characteristics upon initial diagnosis, there was no statistical difference in all-cause mortality among the two groups.

## Supporting information

S1 Table Inclusion and exclusion criteria. (DOCX)

S2 Table Primer sets used in the study. (DOCX)

S3 Table Bone morphogenic protein receptor type 2 mutation among probands and family members. (DOCX)
