# Supplementary material for: Prevalence and clinical features of bone morphogenetic protein receptor type 2 mutation in Korean idiopathic pulmonary arterial hypertension patients: The PILGRIM explorative cohort
Source: PLoS One. 2020 Sep 23;15(9):e0238698. doi: 10.1371/journal.pone.0238698 (PMC7510973; doi:10.1371/journal.pone.0238698)
Supplement: S1 Table — (DOCX) [file pone.0238698.s001.docx]

**Supplementary Material**

**Table S1. Study inclusion and exclusion criteria**

| **Inclusion criteria** |
| --- |
| 1. Patients aged between 20 to 80 years |
| 2. Newly diagnosed IPAH patients: patients who meet the following criteria within 3-months obtained by RHC or echocardiography. RHC criteria: (1) mPAP >25 mmHg at rest and (2) mean PAWP or LVEDP <15 mmHg. Echocardiographic criteria were peak pulmonary arterial pressure (peakPAP) >40mmHg and mPAP >30mmHg. |
| 3. Previously diagnosed patients with WHO category I PAH who were refractory to conventional treatment excluding iloprost inhalation solution (Ventavis™) |
| 4. Patients able to undergo a low-intensity exercise test (bicycling or walking) |
|  |
| **Exclusion criteria** |
| 1. Patients with other left heart disease (WHO pulmonary hypertension category II) e.g. congestive HF, cardiomyopathy, significant valvular heart disease, significant arrhythmia, suspicious elevated PAWP |
| 2. Patients with WHO category III, IV, or V pulmonary hypertension: |
| o    Pulmonary hypertension with lung disease and/or hypoxemia |
| o    Chronic obstructive pulmonary disease |
| o    Interstitial lung disease |
| o    Sleep disorder breathing |
| o    Alveolar hyperventilation disorders |
| o    Chronic exposure to high altitude |
| o    Developmental abnormalities |
| o    Pulmonary hypertension due to chronic thrombotic and/or embolic disease |
| o    Thromboembolic obstruction of the proximal pulmonary arteries |
| o    Thromboembolic obstruction of the distal pulmonary arteries |
| o    Non-thrombotic pulmonary embolism (e.g. tumor or parasitic) |
| o    Miscellaneous disorders affecting the pulmonary vasculature |
| o    Patients with contraindication to Ventavis, hypersensitivity to Ventavis, and high risk of bleeding (e.g. active peptic ulcer, trauma, intracranial hemorrhage) |
| o    Severe coronary disease |
| o    Unstable angina |
| o    History of acute myocardial infarction within 6-months |
| o    Uncompensated heart failure not under close medical monitoring |
| o    Severe arrhythmia |
| o    Suspected pulmonary congestion |
| o    Cerebrovascular disease within 3-months (e.g. transient ischemic attack, stoke) |
| o    Pulmonary hypertension due to venous occlusive disease, valvular defect with dysfunction of cardiac muscle, which is independent of pulmonary hypertension |
| o    Pregnancy |
| o    Women with high probability of pregnancy |
| o    Breast feeding |
| o    Renal failure (creatinine clearance: less than 30mL/min) |
| 3. The patients concurrently using other pulmonary vasodilators (e.g. inhaled NO, endothelin antagonists) except PDE5 inhibitors |
| 4. Patients with poor echo window which is unavailable to accept the echo data |
| 5. Patients unable to do any exercise |
| 6. Patients who change medication during treatment with Ventavis |
| 7. Patients with allergic reaction to Ventavis |
| 8. The patients with other systemic disease (e.g. leukemia, MM, sickle cell anemia, significant liver disease)  9. Pregnant women or prisoners |

Abbreviations: WHO, World Health Organization; PAH, pulmonary arterial hypertension; RHC, right heart catheterization; mPAP, mean pulmonary arterial pressure; LVEDP, left ventricular end-diastolic pressure; peakPAP, peak pulmonary arterial pressure; PAWP, pulmonary arterial wedge pressure; HF, heart failure; NO, nitric oxide; PDE5, phosphodiesterase-5; MM, multiple myeloma
