# Supplementary material for: Prevalence and clinical features of bone morphogenetic protein receptor type 2 mutation in Korean idiopathic pulmonary arterial hypertension patients: The PILGRIM explorative cohort
Source: PLoS One. 2020 Sep 23;15(9):e0238698. doi: 10.1371/journal.pone.0238698 (PMC7510973; doi:10.1371/journal.pone.0238698)
Supplement: S2 Table — (DOCX) [file pone.0238698.s002.docx]

**Table S2. Primer sets used in the study.**

| **exon** | **Primer** | **Sequences** |
| --- | --- | --- |
| 1 | BR2e-1F | 5'-TCAAACTGTATTGTGATACGGGCA -3' |
|  | BR2e-1R | 5'-GACGCATGGCGAAGGGCAAGCACA -3' |
| 2 | BR2e-2F | 5'-GAAATTTATGAAGTCATTCGGATA -3' |
|  | BR2e-2R | 5'-GATTTTAACATACTCCCATGTCCT -3' |
| 3 | BR2e-3F-2 | 5'-CTGTTTCATAGCTTACACGTACTC -3' |
|  | BR2e-3R-2 | 5'-ACGCCTGGCTTCAACCTTGAATGT -3' |
| 4 | BR2e-4F | 5'-TTTGATTATACATGGGTACAGCCT -3' |
|  | BR2e-4R | 5'-TCCATACGTGATACTATTGAGGCT -3' |
| 5 | BR2e-5F | 5'-CTTGCTGCTAATCTTTCTGCAGCT -3' |
|  | BR2e-5R | 5'-TGATTGAATGAAGCTACTGTTCCA -3' |
| 6 | BR2e-6F | 5'-AGCAACAGAGAGCTGTAGCATTCT -3' |
|  | BR2e-6R | 5'-CCTCAAGTGATCCACCTGCCTTAG -3' |
| 7 | BR2e-7F | 5'-TTACTCTTCATGTTAAAGTGAGT -3' |
|  | BR2e-7R | 5'-CGTGGGAAAGCTCTTTCTGTTACA -3' |
| 8 | BR2e-8F | 5'-GTATGTTCATTTCATGTTCAATAG -3' |
|  | BR2e-8R-3 | 5'-TGAGGATAATTGTCCTGAGTCTCA -3' |
| 9 | BR2e-9F | 5'-GGTTAGGGTCAAATAACATTGACA -3' |
|  | BR2e-9R | 5'-AAAGTTGAGTTAGGTACTATAGGT -3' |
| 10 | BR2e-10F | 5'-CCTACAGCAAGGTCTCTTTAGGAT -3' |
|  | BR2e-10R | 5'-GATTTGTGGCATTAGGCAACTCCA -3' |
| 11 | BR2e-11F | 5'-CATGTTCCGTAATCCTTGAAGCCT -3' |
|  | BR2e-11R | 5'-AGGTAATCATTGAACTATTAGGCT -3' |
| 12 | BR2e-12aF | 5'-TAAATGTACGTTCTCAATGTGATA -3' |
|  | BR2e-12aR | 5'-GGCCACTGAACTGTTTAAGAGAGT -3' |
|  | BR2e-12bF3 | 5'-TATATCTGAGATGCCATACCCAGA -3' |
|  | BR2e-12bR3 | 5'-AAGTTTGATTTGTGCTTGCTGCCA -3' |
|  | BR2e-12bF | 5'-CCAAAAGAAGTTGATAAGAACCTC -3' |
|  | BR2e-12dR3 | 5'-CTCACCAATAAACTGATTCTGCAA -3' |
|  | BR2e-12cF | 5'-CCATGCTGCCACAACCCAATATGC -3' |
|  | BR2e-12cR2 | 5'-GGATCATTTACAAAAGTGGTAAAGGT -3' |
| 13 | BR2e-13F2 | 5'-CTCCTGAGACATTGGTTTGACCTT -3' |
|  | BR2e-13R | 5'-AAATAATTTCACTCCATAGGCTTG -3' |
