# Supplementary material for: Prevalence and clinical features of bone morphogenetic protein receptor type 2 mutation in Korean idiopathic pulmonary arterial hypertension patients: The PILGRIM explorative cohort
Source: PLoS One. 2020 Sep 23;15(9):e0238698. doi: 10.1371/journal.pone.0238698 (PMC7510973; doi:10.1371/journal.pone.0238698)
Supplement: S3 Table — (DOCX) [file pone.0238698.s003.docx]

**Table S3. Bone morphogenic protein receptor type 2 mutation among probands and family members.**

| **Identification** | | ***BMPR2* mutation** |
| --- | --- | --- |
| **Patient #1** | **Proband** | YES |
|  | Father | NO |
|  | Mother | YES |
|  | Sister | NO |
| **Patient #2** | **Proband** | YES |
|  | Father | NO |
|  | Mother | YES |
| **Patient #3** | **Proband** | YES |
|  | Daughter | YES |
| **Patient #4** | **Proband** | YES |
|  | Father | YES |
|  | REF | NO |
|  | Sister | YES |
|  | Brother | YES |
|  | Mother | NO |
| **Patient #5** | **Proband** | YES |
|  | Father | YES |
|  | Mother | NO |
| **Patient #6** | **Proband** | YES |
|  | Father | YES |
|  | Mother | NO |
| **Patient #7** | **Proband** | YES |
|  | REF | YES |
|  | REF | YES |
|  | REF | NO |

REF: refused to share
